# The essential role of optical flow in the peripheral visual field for stable quiet standing: Evidence from the use of a head-mounted display

**DOI:** 10.1371/journal.pone.0184552

**Published:** 2017-10-09

**Authors:** Kentaro Horiuchi, Masami Ishihara, Kuniyasu Imanaka

**Affiliations:** 1 Department of Health Promotion Sciences, Tokyo Metropolitan University, Tokyo, Japan; 2 Department of Human Sciences, Tokyo Metropolitan University, Tokyo, Japan; Tokai University, JAPAN

## Abstract

It has long been thought that vision is the most essential factor in maintaining stable quiet standing compared to other sources (i.e., vestibular and somatosensory inputs) of information. Specifically, several vision studies on postural control have shown evidence for the importance of the visual system, particularly peripheral vision rather than central vision, and optical flow. Nevertheless, to date, no study has manipulated both visual field and optical flow concurrently. In the present study, we experimentally manipulated both the visual field (the central and peripheral visual fields) and the occurrence of optical flow during quiet standing, examining the effects of the visual field and optical flow on postural sway measured in terms of the center of pressure (CoP). Stationary random dot stimuli were presented exclusively in either the central or peripheral visual field, while the occurrence of optical flow was manipulated using a desktop (DTD) or a head-mounted (HMD) display. The optical flow that occurred while using the DTD was a function of the postural sway during quiet standing, while for the HMD, no optical flow occurred even when the body/head swayed during quiet standing. Our results show that the extent of postural sway (e.g., CoP area) was smaller when visual stimuli were presented in the peripheral visual field than that in the central visual field; this was the case while using the DTD alone, with no effects of the peripheral vision on the extent of postural sway while using the HMD. It is therefore suggested that the optical flow occurring in the peripheral visual field is essential for stable quiet standing.

## Introduction

Vision generally provides rich information about an individual’s own motion in the environment, and is recognized as an important source of information for motor control, often overriding other sources of information, such as vestibular and somatosensory inputs. Specifically for postural control, two primary points have frequently been discussed. One is the visual field–in particular, the central and peripheral visual fields [1–7]–and the other is optical flow [8–9]; that is, the apparent motion of environmental objects in a visual scene. Optical flow is generally caused by a change in distance between the observer’s eyes and environmental objects in a scene; this distance systematically varies with postural sway [3, 4, 7, 10–20]. These two visual factors that essentially affect postural control have so far not been adequately studied using experimental twofold manipulation of both “visual field” and “optical flow”. Therefore, it is far from clear how the visual field and optical flow affect postural control.

In general, two functional modes of vision, focal and ambient, are distinguishable, with different functional characteristics and underlying information processing. Focal vision is assumed to be responsible for detecting the physical characteristics of environmental objects (that are usually fixated in the central visual field), while ambient vision is concerned with the detection of spatial characteristics of the surrounding (i.e., peripheral) visual world [6]. Studies on visual functions have used various definitions for the central and peripheral visual fields. One neuro-anatomical definition equates central vision with the central 2 to 4° of the visual field, based on the retinal distribution of photoreceptors [21]. Another definition indicates that central vision covers the central 7° of the visual field because visual inputs from the 7° visual field project onto the area of the primary visual cortex that processes central vision [22]. Based on these definitions, Berencsi, Ishihara, and Imanaka [23] used both 4° and 7° visual angles for manipulating the central and peripheral vision conditions. In contrast, Nougier, Bard, Fleury & Teasdale [24, 25], Paulus et al. [18], Brandt et al. [2] and Previc & Neel [26], manipulated the central vision using visual fields of 10°, 30°, and up to 60°, respectively.

Methodologies used to manipulate the visual fields have differed among studies. Nevertheless, a number of studies examining various aspects of postural control in relation to central and peripheral vision have reported a similar finding that postural control during quiet standing was more affected by peripheral vision than central vision [1–7]. For example, Stoffregen [7] and Stoffregen, Schmuckler, and Gibson [27] manipulated the central vision by presenting moving visual stimuli in front of the participants, while manipulating the peripheral vision by presenting the visual stimuli to both the left and right sides of the participants. Their results showed that postural sway was more synchronized to the moving visual stimuli presented in the peripheral visual field than those in the central visual field. However, their visual stimulation might be inadequate to properly examine the respective functional roles of central and peripheral vision in postural control because the visual field of the participant was not directly manipulated, for example, using an occlusion method for the central or peripheral visual field. Hence, the non-occlusion method used by Stoffregen and his colleagues might have resulted in objects other than the presented visual stimuli being present in the participant’s visual field.

In contrast, Berencsi et al. [23] used occlusion manipulation to differentiate the central and peripheral visual fields, presenting stationary random-dot stimuli exclusively in either the central or peripheral visual field. Berencsi et al. measured the trajectory of the center of pressure (CoP) during quiet standing. The CoP area (a primary indicator of the extent of postural sway) was significantly smaller for peripheral vision than that for central vision. This indicated that the visual stimuli in the peripheral rather than central visual field contributed more effectively to enable stable standing. Unfortunately, Berencsi et al. manipulated the visual field alone and did not manipulate the optical flow induced by postural sway. It was therefore far from clear whether the contribution that stabilized posture came from peripheral visual inputs per se or the optical flow likely to occur in the peripheral visual field.

For the manipulation of optical flow, Lee and others [11–16] examined postural sway using a swinging room; the participants stood on the stable floor, surrounded by three-way walls and a ceiling that were oscillated in a forward and backward direction by the experimenter. Their results showed that postural sway was affected by the oscillatory motion of the moving walls and ceiling. This was explained in terms of the optical flow induced by the motion of the walls during quiet standing, such that the systematic variation of the optical flow affected the postural sway even when the participants’ quiet standing had been stable on the fixed floor. Recently, swinging room research has also been conducted with the use of video graphic projection of virtual moving stimuli similar to a real-world swinging room [15–16], and it was confirmed that postural sway was affected by the real-world swinging room as well as the video graphic projection (though with somewhat equivocal results; see the Discussion section). Nevertheless, because these studies manipulated only the optical flow, but did not manipulate the visual field, it was far from clear whether the resultant postural sway was affected by the optical flow occurring in the central or peripheral visual field.

In the present study, CoP during quiet standing was examined while manipulating both the visual field (central and peripheral) and optical flow (its occurrence) to reveal the relative importance of the central/peripheral visual fields and optical flow. The occurrence of optical flow was manipulated using two different types of display, a desktop (DTD) and a head-mounted (HMD) display, with visual random-dot stimuli being presented in either the central or peripheral visual field in an identical manner for both the DTD and HMD conditions. For the DTD condition, optical flow should necessarily occur as a function of body/head sway during quiet standing, whereas no optical flow should occur while using the HMD even when the body/head sways. If visual input per se in the peripheral visual field is essential for postural control (as shown by [23]), CoP variables for peripheral vision should be similar for both the DTD and HMD conditions. In contrast, if optical flow in the peripheral visual field is essential to maintain postural stability, CoP variables should be significantly more stable for the DTD than for the HMD condition.

## Methods

### Participants

Twenty-eight healthy graduate and undergraduate students (9 male and 19 female), aged 23.2 ± 3.1 years, participated in this experiment. They all had normal or corrected-to-normal vision, with no evidence or known history of postural or skeletal disorders. The experiment was approved by the Ethics Committee of Tokyo Metropolitan University, and each participant was informed of the experimental procedures and gave written consent to take part in the experiment.

### Apparatus

#### Experiment room

The experiment was conducted in a completely dark room. Light from outside the room was occluded, and all the apparatus emitting light in the dark room were covered with a black sheet. The reason for using a dark room was that the participants then were able to see only the experimental visual stimuli presented on the display in the DTD condition, eliminating any differences in visual circumstances between the DTD and HMD conditions. Specifically, for the HMD condition, the display was embedded inside the goggle of an HMD; thus the experimental visual stimuli alone were visible. This was replicated for the DTD condition through the use of a completely dark room. The inside of the dark room was monitored using an infrared camera during the experimental trials by the experimenter seated outside the room.

#### Force platform

A force platform (Kistler, 9286B) and an amplifier (Kistler, 3863A) were used to measure the trajectory of CoP during quiet standing. Analog signals of the CoP trajectory were sampled with a frequency of 50 Hz through a 20 Hz low-pass filter and converted into digital data that were subsequently analyzed using a personal computer system (Panasonic, Let’s Note CF-4W). Using analysis software (DKH, TRIAS), five dependent variables, namely, three area variables (envelopment, rectangular, and root mean square areas, cm^2^), total length of CoP displacement (cm), and total length of CoP displacement per area (cm/cm^2^), were calculated from the CoP trajectory data [28–32]. The three area variables indicated the extent of postural sway, irrespective of sway direction. Both the envelopment and the rectangular areas are sensitive to a single/few large deviations of postural sway; this is not the case for the root mean square area variable, which indicates an overall extent of postural sway. The total length of CoP displacement (i.e., the total trajectory length of postural sway) is independent of both the sway direction and area extension. The total length of CoP displacement per area indicates the density of CoP displacement, meaning the extent of dense/immediate postural correction for postural displacement. Below we present the definition of each variable.

Envelopment area (cm^2^)

This variable was calculated through the following procedure:

1. The x-y-plane was divided into 3-degree segments using the center of the sway area (average of the X and Y coordinates) as the central point.
2. In each segment, the data point furthest from the center was selected.
3. Linear interpolation was used to connect neighboring points.
4. The area enclosed by the lines (the envelopment area) was calculated.

$$S_{Env}=\frac{1}{2}\overset{N}{\underset{i=1}{\sum }}\left|X_{i+1}Y_{i}-X_{i}Y_{i+1}\right|$$

*S*_*Env*_ = *Envelopment area*

*X* = {*X*_1_, *X*_2_,…*X*_*N*_}

*Y* = {*Y*_1_, *Y*_2_,…*Y*_*N*_}

*N*: *number of data points*

Rectangular area (cm^2^)

*S*_*Rect*_ = {[*Max*(*X*) − *Min*(*X*)] × [*Max*(*Y*) − *Min*(*Y*)]}

*S*_*Rect*_ = *Reactangular area*

*X* = {*X*_1_, *X*_2_,…*X*_*N*_}

*Y* = {*Y*_1_, *Y*_2_,…*Y*_*N*_}

*N*: *number of data points*

RMS area (cm^2^)

A circular area using the root mean square (RMS) CoP displacement as radius.

$$S_{RMS}=\frac{1}{n}\overset{N}{\underset{i=1}{\sum }}\left|{\left(X_{i}-\bar{X}\right)}^{2}+{\left(Y_{i}-\bar{Y}\right)}^{2}\right|\times \pi$$

*S*_*RMS*_ = *RMS area*

*X* = {*X*_1_, *X*_2_,…*X*_*N*_}

*Y* = {*Y*_1_, *Y*_2_,…*Y*_*N*_}

*N*: *number of data points*

Total length of CoP trajectory (cm)

The sum of displacements between consecutive data points

$$L=\overset{N}{\underset{i=1}{\sum }}\sqrt{\left[{\left(X_{i+1}-X_{i}\right)}^{2}+{\left(Y_{i+1}-Y_{i}\right)}^{2}\right]}$$

*L* = *total length of CoP tragectory*

*N*: *number of data points*

Total length of CoP per area (cm^-1^)

The total CoP trajectory divided by the envelopment area.

LSEnv

Five variables, rather than a single variable, were used to achieve a better assessment of postural sway based on the respective characteristics of each variable. Three trials per condition were performed for each participant, with the mean values of each variable being used as representative data for the subsequent analyses. The raw data of the CoP (anterior-posterior and medio-lateral axes) were visually monitored by the experimenter after every trial, and if a large deviation of postural sway (more than 3 standard deviations) occurred, the data from that trial were discarded from subsequent analyses. In this case, a new trial was performed to give three viable trials for each condition.

#### Display

**Fig 1:**
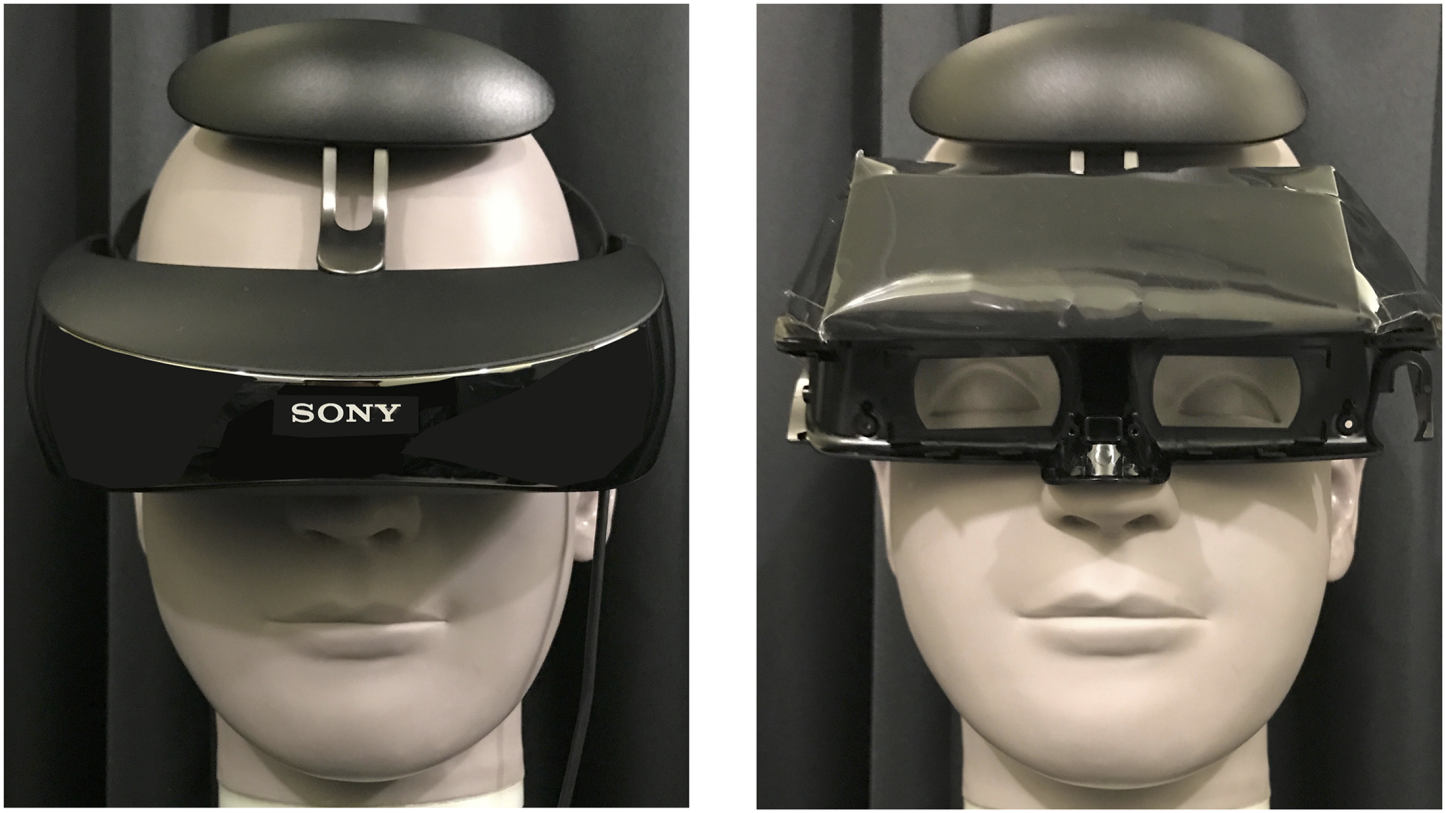
Images of the original and dummy HMDs used. The original (left figure) and dummy (right figure) head-mounted displays (HMD) were respectively worn at HMD and Desktop display (DTD) conditions.

For presenting visual stimuli during quiet standing, two different types of display, DTD (BenQ, ET-0027-B, 27-inch) and HMD (Sony, Personal 3D Viewer, HMZ-T3W; Fig 1, left), were used to manipulate the occurrence (DTD) or non-occurrence (HMD) of optical flow induced by postural sway during quiet standing. Both the DTD and HMD had a resolution of 1290 × 1080 pixels. The visual angles for the HMD were fixed at 45° and 25.5° for the horizontal and vertical axes, respectively. The DTD was set at a viewing distance of 67 cm in order to subtend identical visual angles. The height of the DTD was adjusted for each participant’s eye level. The refresh rate of both the DTD and HMD was 60Hz. While using the DTD, participants wore a dummy HMD (Fig 1, right) during quiet standing. This enabled the participants to perform quiet standing in an equivalent condition of weight load and balance while undergoing both the DTD and HMD assessment. For the dummy HMD, the display monitor unit of an original HMD was replaced with lead weights inside the dummy HMD to maintain the same weight and center of mass as the original HMD. Prior to the experiments, the perceptual similarity of the original and dummy HMDs while wearing them during quiet standing was tested. Ten participants put on the original and the dummy HMDs in a random order and judged the type of HMD they were wearing, with 30 forced-choice trials performed in the dark room. Results showed that 56% of the judgements were correct, with no significant difference from a chance level (*p* > 0.05). This indicated that participants were not significantly aware of any difference during quiet standing.

#### Electrooculography

Electrooculography (EOG) was performed on the right eye to determine the participant’s eye movement during the presentation of visual stimuli. The participants were asked to gaze at a fixation cross, and EOG was used to check the participants’ fixation. For the vertical EOG, a pair of electrodes were placed above and below the right orbit. For the horizontal EOG, an electrode was placed just laterally to the angulus oculi lateralis and the other one medially to the angulus oculi medialis. Analog signals from the electrodes were sampled at 200 Hz and then amplified by an amplifier (NIHON KODEN, Nistagmograph Amplifier, 601G). These EOG data were analyzed with data acquisition software (AD Instruments, Power Lab), with a 30 second interval per trial being rectified, and the mean value of the amplitude defined as the amplitude of eye movement. The EOG data were visually monitored by the experimenter during trials and if a large eye movement occurred, the data from that trial were discarded from subsequent analyses. In this case, a new trial was performed to give three viable trials for each condition.

### Stimuli and visual field conditions

#### Stimuli

The visual stimuli consisted of approximately 3,000 random dots (8 pixels in diameter), generated and controlled by software (Presentation, Neurobehavioral Systems) run on a computer (Diginnos, Raytrek LC-M) and presented on the display monitor (DTD or HMD). A luminous green cross (0.68° in width and height) was used as the fixation point, presented at the center of each display monitor throughout the experimental trials. Stationary white random dots were presented on a black background for both the DTD and HMD, with no dynamic changes in the appearance of the white random dots for any trial or experimental condition.

#### Visual field conditions

**Fig 2:**
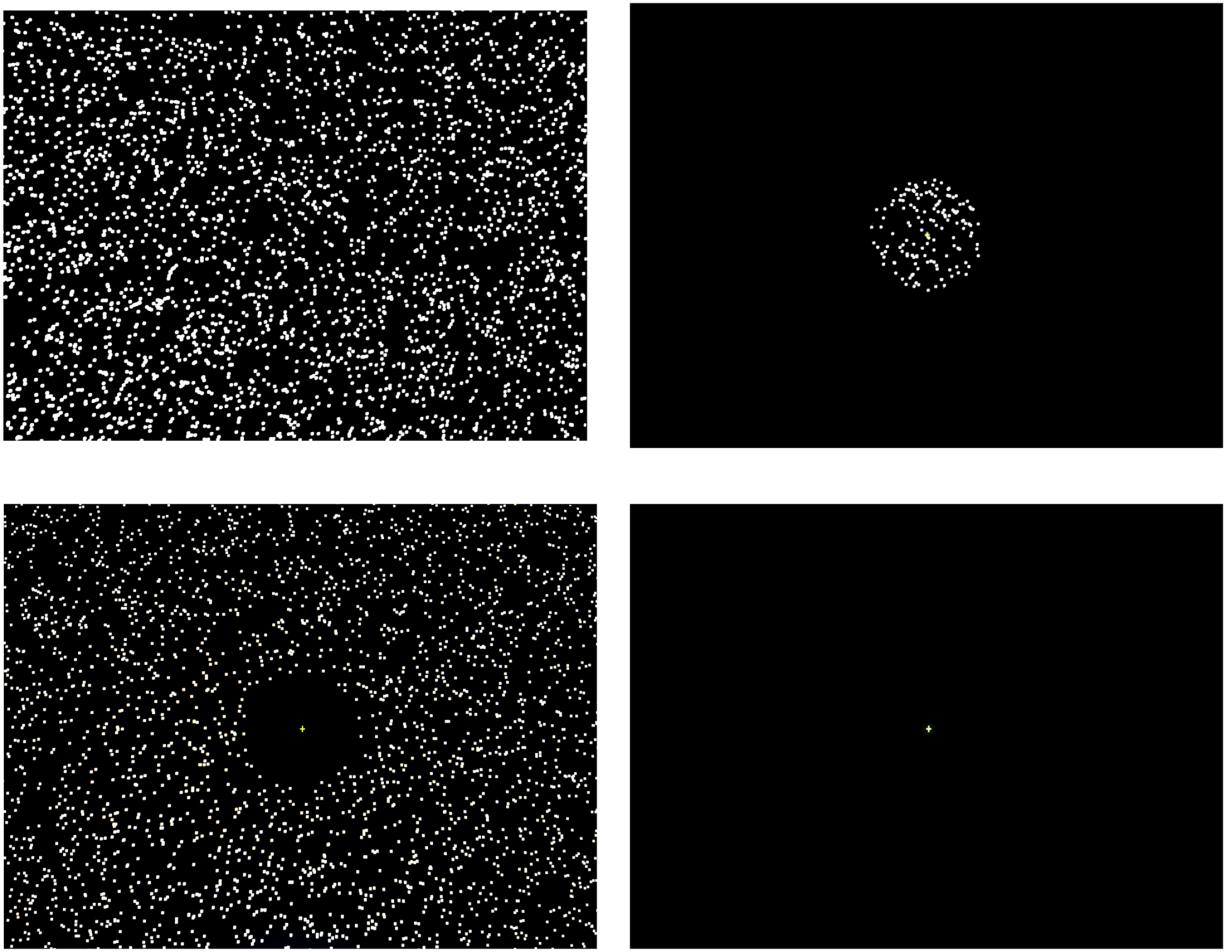
Sample images of the visual stimuli. Sample images of visual stimuli at four visual field conditions (full vision [FV], central vision [CV], peripheral vision [PV], and no dot [ND] condition) presented at the desktop display (DTD) and head-mounted display (HMD) conditions.

Four visual field conditions were experimentally manipulated during quiet standing: full vision (FV), central vision (CV), peripheral vision (PV), and no dot (ND) condition (Fig 2). Both the green fixation cross at the center of the display and the random dot pattern in the full display (i.e., 45° wide and 25.5° high) were presented with or without occlusion of the central or peripheral visual field. For the FV condition, the random dot pattern and fixation cross were presented with no occlusion. For the CV condition, the random dot pattern in the area outside the central visual field of approximately 8° (c.f., [23]) in diameter was occluded, whereas for the PV condition the area inside the central visual field was occluded, with the fixation cross being presented. For the ND condition, the fixation cross alone was presented with no random dot pattern. Quiet standing was performed under these four visual field conditions for both DTD and HMD display conditions.

### Procedure

The participants were asked to perform quiet standing for 60 s on the force platform in Romberg’s standing posture (i.e., both bare feet placed side by side with no gap) and arms relaxed at either side of the body. They were asked to stare at the green fixation cross presented at eye level in front of them throughout the quiet standing trials. Quiet standing was performed under the four visual field conditions (FV, CV, PV, and ND). Three trials of 60 s quiet standing were performed per visual field condition, resulting in 12 trials per display condition (DTD and HMD), and a total of 24 trials per participant. The order of both the four visual field conditions and the two display conditions were counter-balanced among participants. Participants had a 1 min break after every trial, with a long (about 5 to 10 min) break being provided between the HMD and DTD conditions. The total experiment time was around 90 min. We used CoP data collected in the range of 15 to 45 s during the 60 s standing trial for subsequent analyses. The first 15 s period of standing was considered a “settlement period”, in which a relatively large postural sway tended to occur (shown in our preliminary examination). For the last 15 s of standing, it was likely that participants anticipated the end of the trial and this could potentially influence postural control. Upon completion of the whole experiment, participants were asked to write a face sheet, and then asked to verbally report their general impression about the quiet standing trials, easy/difficult visual field conditions, any difference between the DTD and HMD conditions, etc. Subsequent categorization of the participant verbal reports showed small individual variations but no general tendencies, and there were no reports of motion sickness in any condition.

### Data analyses

Two-way (visual field × display) repeated measures ANOVAs were separately performed on both the five CoP variables and the horizontal/vertical EOG variables. When significant interactions were found, simple main effects tests would subsequently be performed; for significant main effects, multiple comparisons would be performed by the Bonferroni method. Statistical significance (*α* = 0.05) was corrected by the Bonferroni procedure, resulting in a corrected alpha of 0.01 for the CoP variables and 0.025 for the EOG variables. For each significant main effect, the effect size was calculated using partial eta squared (*η*_*p*_^*2*^*)*.

## Results

### CoP variables

**Fig 3:**
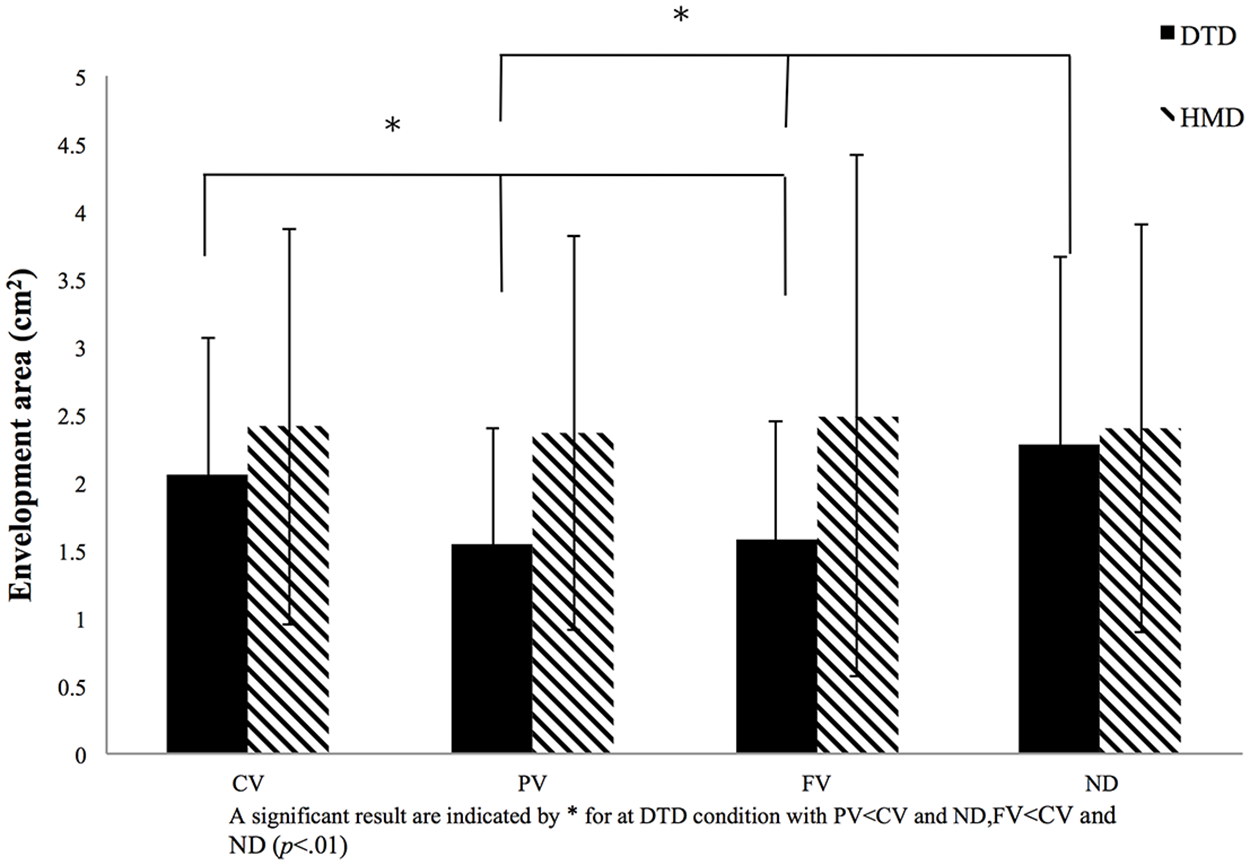
Results for envelopment area. Envelopment area for the four visual conditions (full vision [FV], central vision [CV], peripheral vision [PV], and no dot [ND] condition) for both the desktop display (DTD) and head-mounted display (HMD) conditions. The x-axis denotes the visual condition, with the y-axis denoting the size of the envelopment area. Error bars indicate the standard deviation (SD).

ANOVAs were performed separately on the five CoP variables, resulting in significant interactions between display and visual field for all variables, with significant main effects differing among the five variables. The most clear results were seen for the envelopment area (Fig 3). There were significant main effects for both display [*F* (1, 27) = 9.98, *p* < 0.01, *η*_*p*_^*2*^ = 0.24] and visual field [*F* (3, 81) = 5.86, *p* < 0.01, *η*_*p*_^*2*^ = 0.21], with significant interaction between display and visual field [*F* (3, 81) = 514.41, *p* < 0.001, *η*_*p*_^*2*^ = 0.21]. Subsequent simple main effects tests showed a significant simple main effect for visual field in the DTD condition [*F* (3, 81) = 13.52, *p* < 0.001, *η*_*p*_^*2*^ = 0.32], with PV and FV conditions showing significantly smaller envelopment areas than both CV and ND conditions (*p* < 0.05). In contrast, for the HMD condition, there was no significant simple main effect for visual field [*F* (3, 81) < 1.0]. This indicated that the envelopment area did not significantly differ for the four visual field conditions when stimuli were presented on the HMD (where no optical flow occurred).

**Fig 4:**
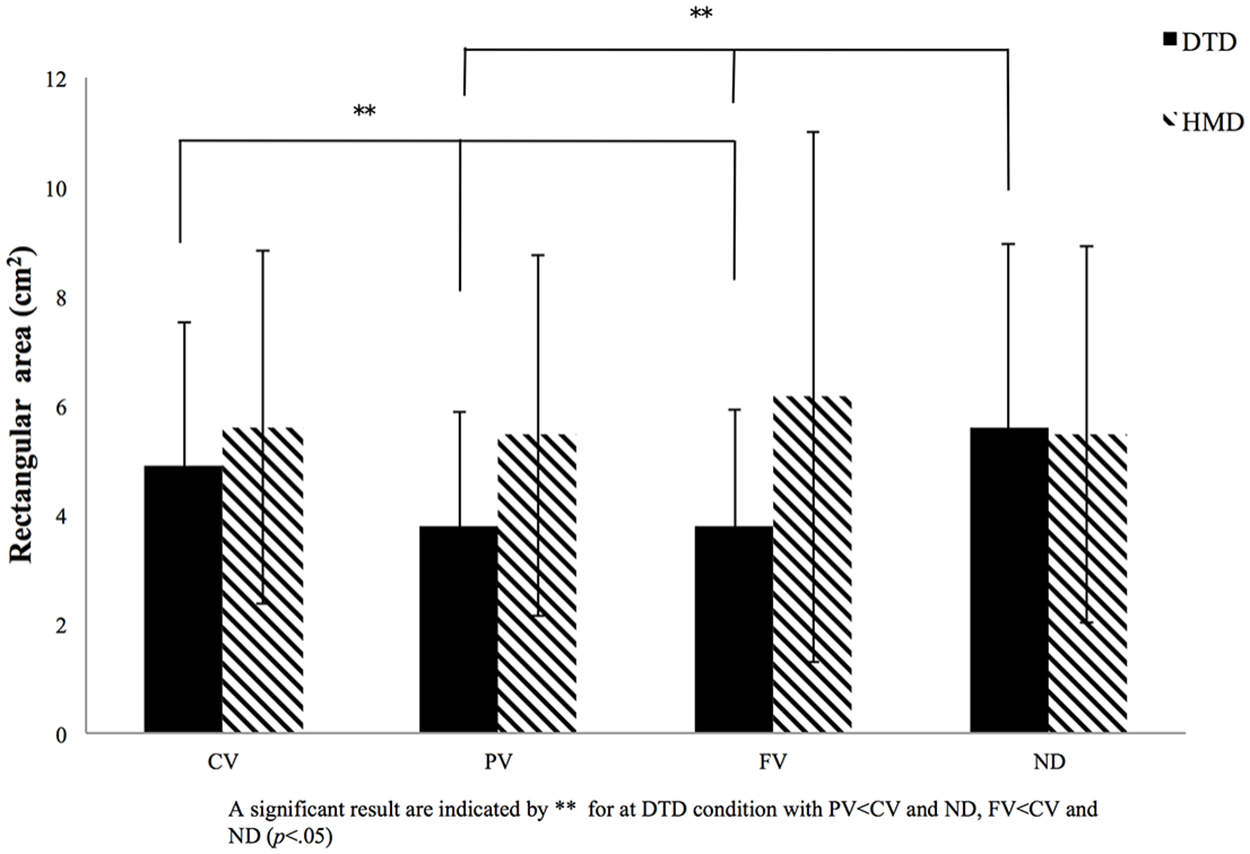
Results for rectangular area. Rectangular area for the four visual conditions (full vision [FV], central vision [CV], peripheral vision [PV], and no dot [ND] condition) for both the desktop display (DTD) and head-mounted display (HMD) conditions. The x-axis denotes the visual condition, with the y-axis denoting the size of the envelopment area. Error bars indicate the standard deviation (SD).

**Fig 5:**
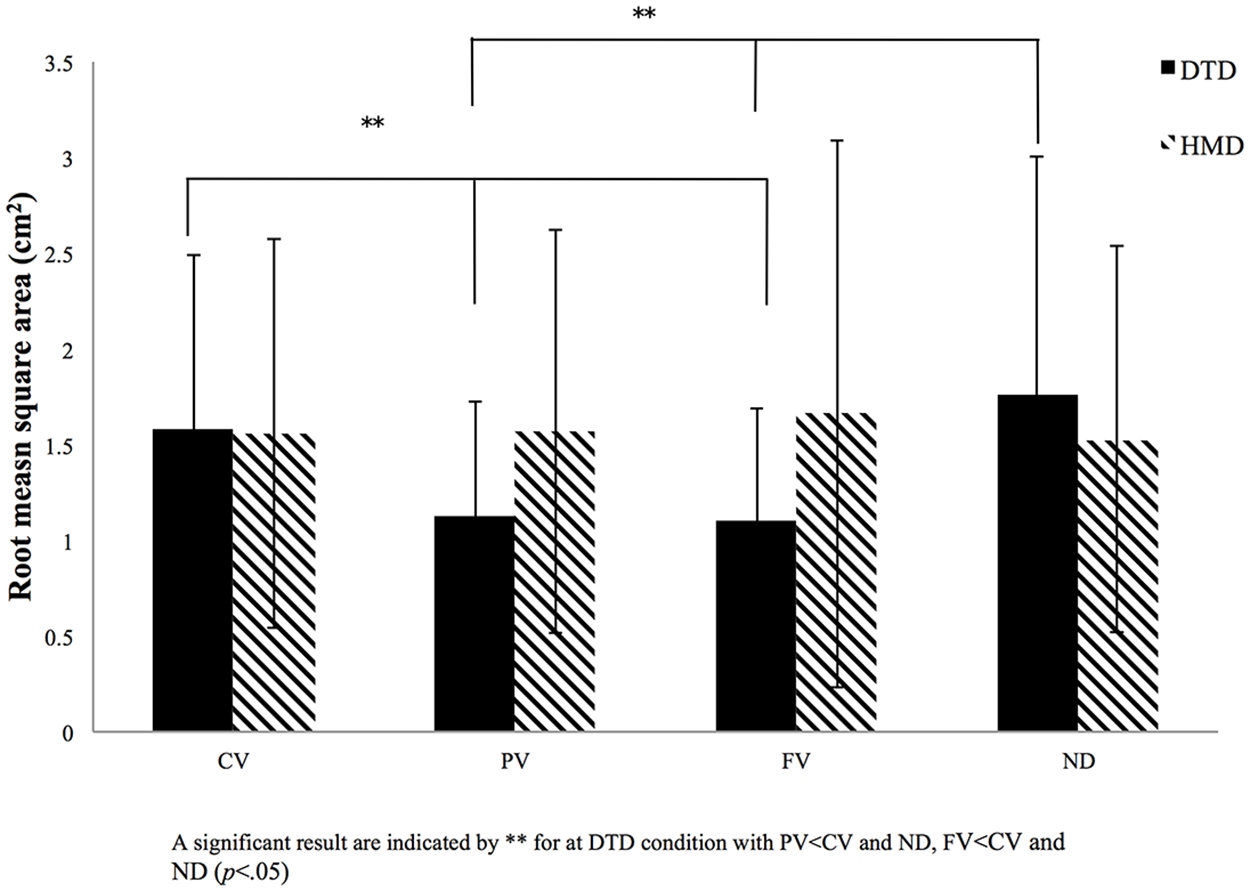
Results for root mean square area. Root mean square area for the four visual conditions (full vision [FV], central vision [CV], peripheral vision [PV], and no dot [ND] condition) for both the desktop display (DTD) and head-mounted display (HMD) conditions. The x-axis denotes the visual condition, with the y-axis denoting the size of the envelopment area. Error bars indicate the standard deviation (SD).

**Fig 6:**
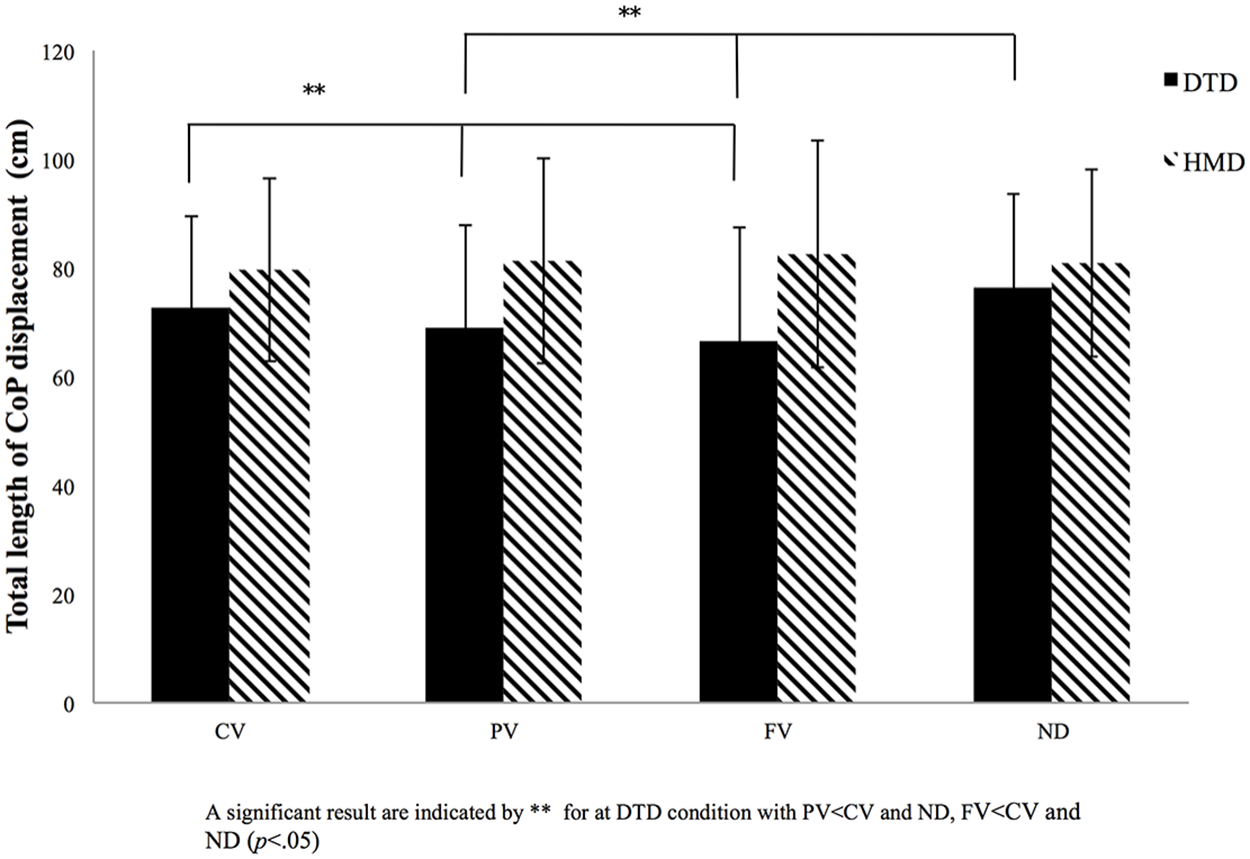
Results for total CoP length. Total length of center of pressure (CoP) displacement for the four visual conditions (full vision [FV], central vision [CV], peripheral vision [PV], and no dot [ND] condition) for both the desktop display (DTD) and head-mounted display (HMD) conditions. The x-axis denotes the visual condition, with the y-axis denoting the total length of CoP displacement per area. Error bars indicate the standard deviation (SD).

**Fig 7:**
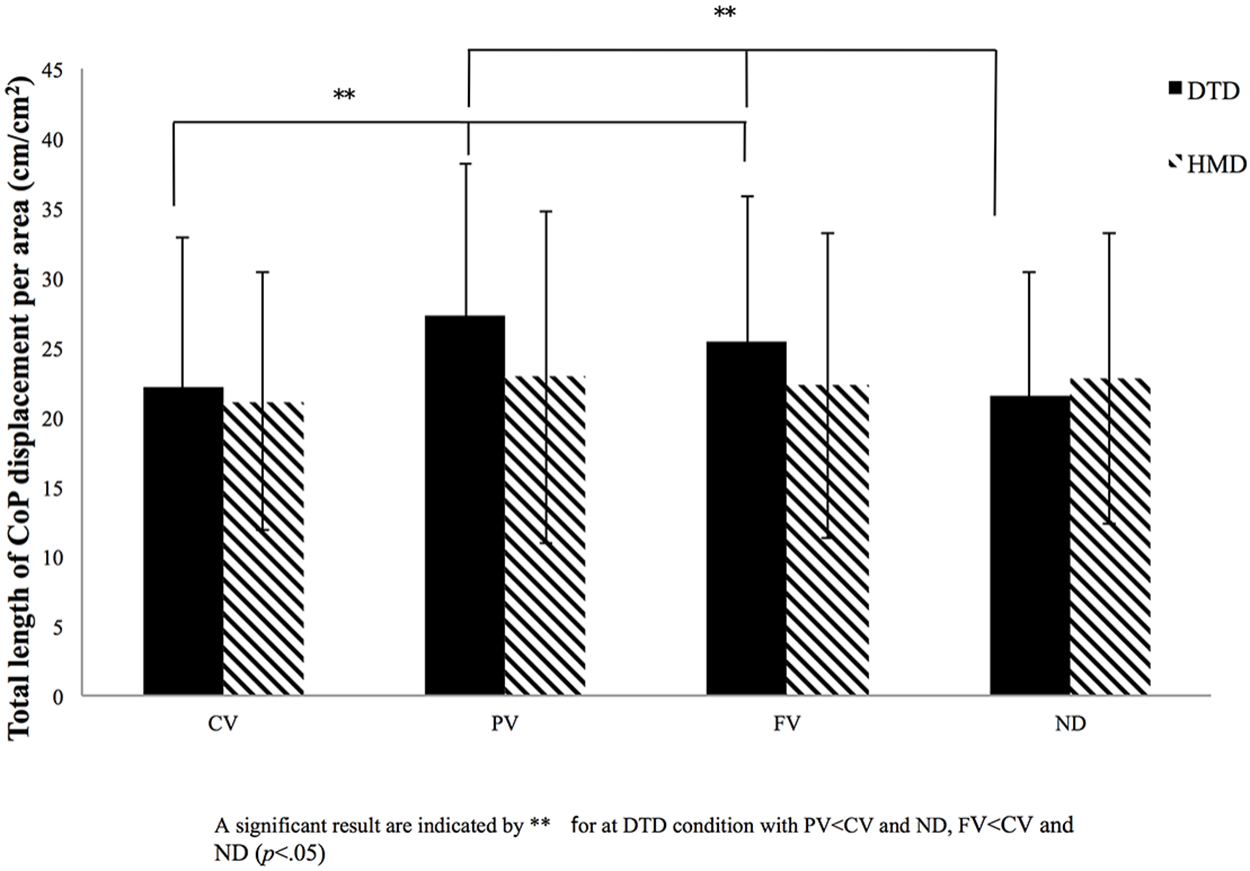
Results for total CoP length over displacement area. Total length of center of pressure (CoP) displacement per area for the four visual conditions (full vision [FV], central vision [CV], peripheral vision [PV], and no dot [ND] condition) for both the desktop display (DTD) and head-mounted display (HMD) conditions. The x-axis denotes the visual condition, with the y-axis denoting the total length of CoP displacement per area. Error bars indicate the standard deviation (SD).

The other four variables–rectangular area, root mean square area, total length of CoP displacement, and total length of CoP displacement per area–showed some differences for both main effects and significant interactions (Figs 4–7). Interactions between display and visual field were significant for rectangular area [*F* (3, 81) = 6.69, *p* < 0.001, *η*_*p*_^*2*^ = 0.22], root mean square area [*F* (3, 81) = 6.82, *p* < 0.001, *η*_*p*_^*2*^ = 0.21], total length of CoP displacement [*F* (3, 81) = 8.24, *p* < 0.001, *η*_*p*_^*2*^ = 0.22], and total length of CoP displacement per area [*F* (3, 81) = 4.68, *p* < 0.001, *η*_*p*_^*2*^ = 0.14]. Subsequent simple main effects tests for the visual field using DTD showed significant simple main effects for all the four variables (rectangular area, *F* (3, 81) = 10.24, *p* < 0.001, *η*_*p*_^*2*^ = 0.26; root mean square area, *F* (3, 81) = 8.94, *p* < 0.001, *η*_*p*_^*2*^ = 0.24; total length of CoP displacement, *F* (3, 81) = 8.16, *p* < 0.001, *η*_*p*_^*2*^ = 0.22; and total length of CoP displacement per area, *F* (3, 81) = 9.85, *p* < 0.001, *η*_*p*_^*2*^ = 0.25). Subsequent multiple comparison tests performed to compare the four visual fields in the DTD condition showed that rectangular area, root mean square area, and total length of CoP displacement were significantly (*p* < 0.05) smaller, and the CoP displacement per area was significantly larger for PV and FV conditions than for CV and ND conditions. In the HMD condition, no significant simple main effects for the visual field appeared for any of the four CoP variables (*F* (3, 81) < 1.0). These results of significant interactions and post hoc simple main effects tests and multiple comparisons are the same as those in the envelopment area depicted in Fig 3.

A recent study has reported differences in response between male and female participants in virtual reality (VR) settings [33]. To check for any such effect of sex in our results, we performed three-way ANOVAs (sex × visual field × display) on each of five dependent variables. This resulted in no significance for either main effects or interaction relating to sex, thus indicating that the results from the CoP data were not significantly affected by sex.

### EOG variables

For EOG displacements in both vertical and horizontal directions, no significant main effects appeared for display [*F* (1, 27) < 1.0] or visual field [*F* (3, 81) < 1.0], with no significant interaction between the two [*F* (3, 81) < 1.0]. This indicated that the eye displacements in both vertical and horizontal directions did not significantly differ between all the four visual field and two display conditions.

## Discussion

The primary focus of this study was to examine the influence of the visual field and optical flow on postural control. The results showed a significant interaction between visual field and display, with a significant simple main effect for the visual field present only in the DTD condition. Specifically, in the DTD condition, the three CoP area variables (i.e., envelopment area, rectangular area, and root mean square area) and the total length of CoP displacement were significantly smaller, and the total length of the CoP displacement per area was significantly larger for the PV and FV conditions than for the CV and ND conditions. In contrast, in the HMD condition, no significant differences were found among the four visual field conditions for any CoP variable. These results thus showed that a smaller CoP area with a larger trajectory displacement of the CoP sway per area was evident for the PV and FV for the DTD condition alone, which is consistent with the previous findings of the Berencsi et al. study [23].

Both the PV and FV conditions for the DTD involved the presentation of visual stimuli in the peripheral visual field with an optical flow due to postural sway occurring in quiet standing. In contrast, no postural advantage appeared at the PV and FV conditions for the HMD, suggesting that visual inputs in the peripheral visual field per se do not affect stable quiet standing unless optical flow is present. As the features of postural sway did not significantly differ between the CV and ND conditions for the DTD, the visual stimuli presented in the central visual field might not contribute to stable quiet standing, even if optical flow is present. These results therefore clearly suggest that the optical flow in the peripheral visual field contributes to better/stable postural control in quiet standing.

With respect to the experimental manipulation of optical flow, in contrast to our experimental setup, most previous studies (e.g., [11–20]) have used dynamic visual stimuli. There are two main ways in which the optical flow is manipulated: (1) the use of a swinging room [11–13, 15, 16] and (2) the presentation of dynamic visual stimuli on a display [14–20]. In both cases, postural response to the visual stimuli is measured. The benefit of such studies is that researchers can experimentally control several aspects of the visual stimuli, such as the amount, pattern, amplitude and frequency, allowing the stimuli to be manipulated within ideal conditions. Depending on the type of visual stimuli used, the researchers can isolate and test different conditions. Nevertheless, such experimental environments also come with a number of limitations that need to be considered. For example, when using dynamical stimuli to control optical flow the visual stimuli on the retina are changed as the result of external motion. However, at the same time optical flow will arise through the postural sway of the participants themselves. In the present study, we employed stationary (rather than dynamic) visual stimuli presented on both a DTD and an HMD, manipulating the existence/non-existence of optical flow due to postural sway alone. We thus showed direct evidence, with no influence of factors related to dynamic visual stimuli, that postural control is mediated by the optical flow perceived in the peripheral visual field.

In addition, with no difference in the amplitude of eye movement for any visual field or display condition, the EOG results indicate that the finding of excellent postural control in the PV and FV conditions for the DTD trials had no relationship with eye movement. Kelly et al. [17] showed that retinal flow stabilizes standing posture in both real world and virtual environments. Retinal flow arises from both head-centric optical flow and rotations of the eye with respect to the head, but our CoP data are free from the influence of eye movements/rotations. In the present study, the EOG variables showed that eye movements were not an influential factor in maintaining stable standing.

The essential role of optical flow for stable postural control in quiet standing has been explained in terms of postural sway arising to minimize the optical flow induced by the swinging room environment that provides dynamic visual stimuli [12, 13, 34]. This is generally supported by the Kelly et al. study [17], in which stationary visual stimuli were overlapped with an altering random dot pattern (no optical flow condition) or not (optical flow condition) in a VR setup. Although the present study also used a stationary random dot pattern, the manipulation for the existence/non-existence of the optical flow was different from the Kelly et al. study. We used exactly the same random dot pattern in both the DTD (providing optical flow) and HMD (no optical flow) conditions. Our results therefore provide direct evidence for the essential role of optical flow for stable standing. Seemingly, a semi-automatic process corrects the postural sway that cancels/minimizes the induced optical flow; thus, postural control in quiet standing seems to be mediated by the use of information derived from the optical flow (which was manipulated in the present study by the use of both the DTD and HMD conditions) (e.g., [10, 35]). Because the distance between the retina and visual environmental objects becomes smaller when the body leans forward, the optical flow expands on the retina. To cancel/minimize such an expansion of the optical flow, a backward sway in the standing posture will be induced. Conversely, when the body tilts backward, the optical flow from contracting images of environmental objects will cause a reaction leading to a forward postural sway. Thus, postural disturbance during quiet standing will be semi-automatically amended by a corrective postural sway, which may be a function of both the direction and amplitude of the optical flow induced.

There are at least two possible reasons why the optical flow in the peripheral field may contribute stronger to achieving stable standing than the one in the central visual field. One is that the visual function of optical flow might differ for the central and peripheral visual fields. It has long been well known that the distribution ratios of the rod and cone photoreceptor cells clearly differ for the central and peripheral visual fields [36]. The visual function of central vision is generally characterized by a high spatial frequency, contributing to an optimization of form vision [37]. On the other hand peripheral vision is characterized by a high temporal frequency and therefore more sensitive to movement [38]. This might be the reason for the importance of optical flow in the peripheral rather than the central visual field in postural control of quiet standing.

A second reason might be a difference in the magnitude of the expansion/contraction of the optical flow between the central and peripheral visual field. The amplitude of the optical flow from postural sway increases with visual angle. As the visual angle of an environmental object from a fixed position is larger in the peripheral visual field than in the central visual field, this indicates that the amplitude of the optical flow induced by postural sway would necessarily be larger in the peripheral visual field than in the central visual field. Therefore, the effects of optical flow on postural control may well be larger for the peripheral visual field than for the central field during quiet standing. This is evident from our results that showed more stable CoP sway for the PV and FV conditions than for the CV and ND conditions in the DTD condition. Such an explanation seems to be consistent with some previous findings, which showed that the postural sway increased as the distance to the visual stimuli increased [39]. This is because the optical flow becomes smaller if a visual stimulus is presented at a more distant position from the observer, resulting in a larger postural sway due to the decreased magnitude of the optical flow. Conversely, if the distance of the visual stimulus to the observer is closer, the optical flow becomes larger, resulting in enhanced postural stability. This is consistent with our explanation of the larger magnitude of the optical flow in the peripheral visual field compared to the central visual field. The result is a more stabilizing effect on postural sway from the optical flow occurring in the peripheral visual field than the central one.

Finally, our study has shown a new application for the use of an HMD and DTD with the presentation of identical visual stimuli in examining the features of postural control with respect to optical flow. The use of an HMD has largely been limited to VR studies [17, 40–43] and few studies have focused on the influence of optical flow induced by postural sway without using dynamical stimuli, such as those in the swinging room paradigm. Kelly et al. [17] used an HMD to show 3D visual stimuli using a stationary image of an environment and manipulated the existence/non-existence of optical flow. For the optical flow condition, the stimulus was a stationary photorealistic image of the environment, while for the no optical flow condition, a generated 3D stationary mesh was overlapped with a random-dot stereogram that changed in every frame of the visual stimulus presentation. With this manipulation, the changing random-dot stereograms eliminated optical flow. However, the optical-flow and the no-optical-flow conditions differed in the visual stimuli presented. Therefore, the results from these two conditions are equivocal regarding the effects of optical flow (rather than the effects of different stimuli) on postural sway. In contrast, we used the same stationary visual stimuli (random dot pattern) presented on two different displays (the DTD and HMD) to manipulate the existence/non-existence of optical flow, thus enabling us to explain the resultant postural stability in terms of the existence/non-existence of optical flow. In addition, the use of both DTD and HMD conditions with identical visual stimuli allowed us to concurrently manipulate the peripheral and central visual field conditions as well. Consequently, in this study, we confirmed that we could manipulate the occurrence of optical flow using an HMD (and DTD for comparison) while presenting stationary visual stimuli.

## Conclusion

The present study examined the effects on CoP in quiet standing of visual random dot stimuli presented in either the central or peripheral visual field (manipulating visual fields) in both DTD (with optical flow) and HMD (no optical flow) display conditions. Our results showed that for the DTD condition, the CoP areas and the total length of CoP displacement were significantly smaller, and the total length of CoP displacement per area was significantly larger, for PV and FV visual field conditions than for CV and ND. In contrast, there were no significant differences among the four visual field conditions for HMD. It is therefore suggested that the optical flow in the peripheral visual field contributed to a better stabilization of postural sway in quiet standing and that unless optical flow occurred, visual stimuli in the peripheral visual field would not contribute to better postural control. From a methodological perspective, the present study clearly shows that the use of an HMD was effective in eliminating the optical flow normally induced during quiet standing.
